# Advocating for language equity: a community-public health partnership

**DOI:** 10.3389/fpubh.2023.1245849

**Published:** 2023-10-17

**Authors:** Dana Kurlander, Amy G. Lam, Elizabeth Dawson-Hahn, Diego de Acosta

**Affiliations:** ^1^Korean Community Center of the East Bay, San Leandro, CA, United States; ^2^National Resource Center for Refugees, Immigrants, and Migrants, University of Minnesota, Minneapolis, MN, United States; ^3^Department of Pediatrics, University of Washington, Seattle, WA, United States

**Keywords:** advocacy and policy, language access, refugees and immigrants, community-based organizations, COVID-19, public health partnership, coalitions, data disaggregation

## Abstract

In the United States, 21.5% of individuals aged 5 or older speak a language other than English at home and 8.2% have Limited English Proficiency (LEP). LEP individuals experience healthcare disparities, including lower access to healthcare services, poorer health outcomes, and higher levels of uninsurance. The COVID-19 pandemic highlighted and exacerbated these health disparities and unmet healthcare needs. In Alameda County (CA), where 46% of foreign-born residents speak a language other than English at home, community-based organizations have been crucial in providing translated materials and one-on-one support to ensure LEP residents receive critical COVID-19 updates and services. Refugee and Immigrant Collaborative for Empowerment (RICE) is a multilingual coalition of seven Alameda County community-based organizations led by the Korean Community Center of the East Bay (KCCEB). During the COVID-19 pandemic, RICE expanded its public health role to fill service and information gaps, advocate on behalf of LEP groups, and build a linguistically and culturally responsive public health safety network. This community case study describes a three-part advocacy-focused intervention that RICE undertook from September 2021 to October 2022. It included (1) a community needs survey, (2) a landscape assessment of the Alameda County Health Department’s (ACPHD) communication materials and online platforms, and (3) relationship building with the ACPHD. The community survey revealed differences across LEP subgroups and highlighted the importance of gathering data disaggregated by language preference. The landscape assessment allowed RICE to understand the ACPHD’s decision-making process and develop data-informed advocacy requests on behalf of LEP communities. Effective communication and coordination between RICE and the ACPHD shortened the feedback loop between public health authorities and LEP communities and laid the groundwork for the RICE organizations to be part of the ACPHD’s future decision making. Data disaggregation, language equity-based advocacy, and cross-sector collaboration were critical ingredients in RICE’s intervention. RICE’s partnership and relationship of mutual accountability with the ACPHD may provide a useful model for other community-based organizations and public health departments seeking to form similar partnerships.

## 1. Introduction

In the United States, 21.5% (67.8 million) of individuals aged 5 or older speak a language other than English at home (1) and 8.2% (25.9 million) have Limited English Proficiency (LEP), meaning they report speaking English less than “very well” (1, 2). Under federal law, healthcare providers who receive federal funds must provide interpreter services free of charge to LEP individuals (2). However, LEP individuals continue to experience healthcare disparities, including lower access to services, poorer health outcomes, limited trust and less effective patient-provider communication, higher levels of uninsurance, and more gaps in health insurance coverage (3–5).

The COVID-19 pandemic highlighted and exacerbated existing health disparities and unmet healthcare needs among LEP individuals (6, 7). LEP individuals often missed out on crucial COVID-19 information because of: (1) insufficient or inadequate translation, (2) lack of culturally congruent and community-specific messaging; and (3) impractical or ineffective communication channels (8). For example, a 2022 study of federal and state COVID-19 vaccine websites found that only 56% offered professionally translated information about vaccine safety and efficacy and only 50% had professionally translated information about how to get the COVID-19 vaccine in at least one language; moreover, translations were often limited in scope and not provided in enough languages to serve local communities (9).

Many public health departments struggled not only to develop accessible messaging, but also to gather and report timely, relevant COVID-19 data for high-risk populations (10). To understand how public health emergencies like COVID-19 affect LEP individuals, these departments need demographic data disaggregated by language preference, race/ethnicity, and country of birth (11, 12). Capturing this level of detail–a prerequisite for planning appropriate interventions–requires cross-sector partnerships between public health and community organizations, along the lines envisioned in the Public Health 3.0 framework (13, 14).

In this article, we describe a collaboration between seven community-based organizations in Alameda County, California, and their partnership with the county public health department. We begin by providing regional context and describing the collaboration between the organizations. Next, we present three programmatic elements that comprised the organizations’ language equity advocacy work: assessing community needs, understanding the public health department’s processes and services, and relationship-building with the public health department. We then evaluate the effectiveness of the three elements, individually and collectively. Finally, we explore how the results of the organizations’ work align with and advance previous studies on data-driven advocacy and cross-sector collaborations.

## 2. Context

Alameda County is the fourth most ethnically, culturally, and linguistically diverse county in the United States. Of the county’s 1.6 million residents, 34.3% are foreign-born, compared to 26.6% for the state of California and 13.6% for the United States as a whole (15). Among foreign-born residents, 46% speak at least one of Alameda County’s 130 languages other than English at home (16, 17). The five most commonly spoken languages after English are Spanish (253,280 speakers), Chinese (145,606), Tagalog (56,869), Vietnamese (29,341), and Korean (14,007) (18). An estimated 8% (46,520) of county households are LEP (19). Historically, Alameda County’s LEP residents have faced significant adverse social conditions, including poverty and overcrowded housing, as well as limited access to transportation, digital technology, and essential services.

The COVID-19 pandemic disproportionately impacted LEP communities in Alameda County for many reasons, including linguistic and cultural barriers to accessing COVID-19 services. At the beginning of the pandemic, county health services across the San Francisco Bay Area, such as COVID-19 websites, communication materials, and clinic testing and vaccination services, were often solely available in English due to limited funding and staffing (20). LEP communities, facing insufficient in-language COVID-19 information and abundant misinformation, looked to networks outside of medical care for support and guidance. Community-based organizations stepped in to bridge this gap, providing translated materials and one-on-one support to ensure the county’s 258,500 LEP residents could receive critical, time-sensitive prevention and treatment updates and use COVID-19 services (15).

In November 2022, Alameda County reported 356,670 cumulative COVID-19 cases, 2,067 deaths, and 1,417,230 fully vaccinated individuals (21). However, the Alameda County Public Health Department’s (ACPHD) COVID-19 website dashboard provided data disaggregated only by race, gender, and age, so it was impossible to see the pandemic’s overall impact on LEP groups or differences across LEP groups.

The Korean Community Center of the East Bay (KCCEB) is a community organization in San Leandro, California that serves Korean and other immigrants in the Bay Area through access to education, services, resources, and advocacy. KCCEB leads and participates in the Refugee and Immigrant Collaborative for Empowerment (RICE), a multiethnic, multilingual coalition among seven community organizations. RICE includes KCCEB, Burma Refugee Families and Newcomers, Center for Empowering Refugees and Immigrants, Diversity in Health Training Institute, Filipino Advocates for Justice, Mental Health Association for Chinese Communities, and Refugee and Immigrant Transitions. Collectively, RICE has over 95 years’ experience building trusted relationships with local refugee and immigrant communities, partnering with each other, and working with Alameda County through previous projects, including 2020 Census Engagement. RICE values working as a multiracial, multilingual collaborative and prioritizes a language equity framework to strengthen its unified voice and address a root cause of disparities in LEP communities.

At the beginning of the COVID-19 pandemic, RICE mobilized to support COVID-19 testing and increase vaccine awareness and accessibility across 16 language groups (Amharic, Arabic, Burmese, Cantonese, English, Farsi, Khmer, Korean, Mam, Mandarin, Nepali, Spanish, Tagalog, Tibetan, Tigrinya, and Vietnamese). At the height of the vaccine rollout, KCCEB received a grant from the National Association of County and City Health Officials (NACCHO) as part of a national effort to support community organizations and health departments developing COVID-19 prevention and mitigation strategies for immigrant communities (22). Through this grant, RICE collaborated with the ACPHD to build a robust public health safety network that would be linguistically and culturally responsive to underserved LEP communities. Between September 2021 and October 2022, KCCEB and RICE expanded their public health role by building bridges among the ACPHD, community organizations, and residents to uplift the voices of LEP groups.

## 3. Key programmatic elements

RICE’s COVID-19 intervention and partnership with the ACPHD, made possible by the NACCHO grant, included direct service and advocacy efforts. The RICE organizations’ approach was inspired by the MAP-IT framework (Mobilize, Assess, Plan, Implement, Track) from the U.S. Department of Health and Human Services’ *Healthy People 2020* program (23, 24). The MAP-IT framework is a step-by-step method to create and evaluate community health interventions.

KCCEB led grant writing and intervention development in close collaboration with leaders from each RICE organization. The RICE organizations then implemented the intervention within their own communities, informed by the National Resource Center for Refugees, Immigrants and Migrants’ *Community Health Workers* promising practice (25). Having employed this model before, RICE’s bilingual and bicultural community health workers were uniquely positioned to combat medical and government mistrust and COVID-19 misinformation, and bridge linguistic and cultural gaps for the ACPHD.

**Figure 1:**
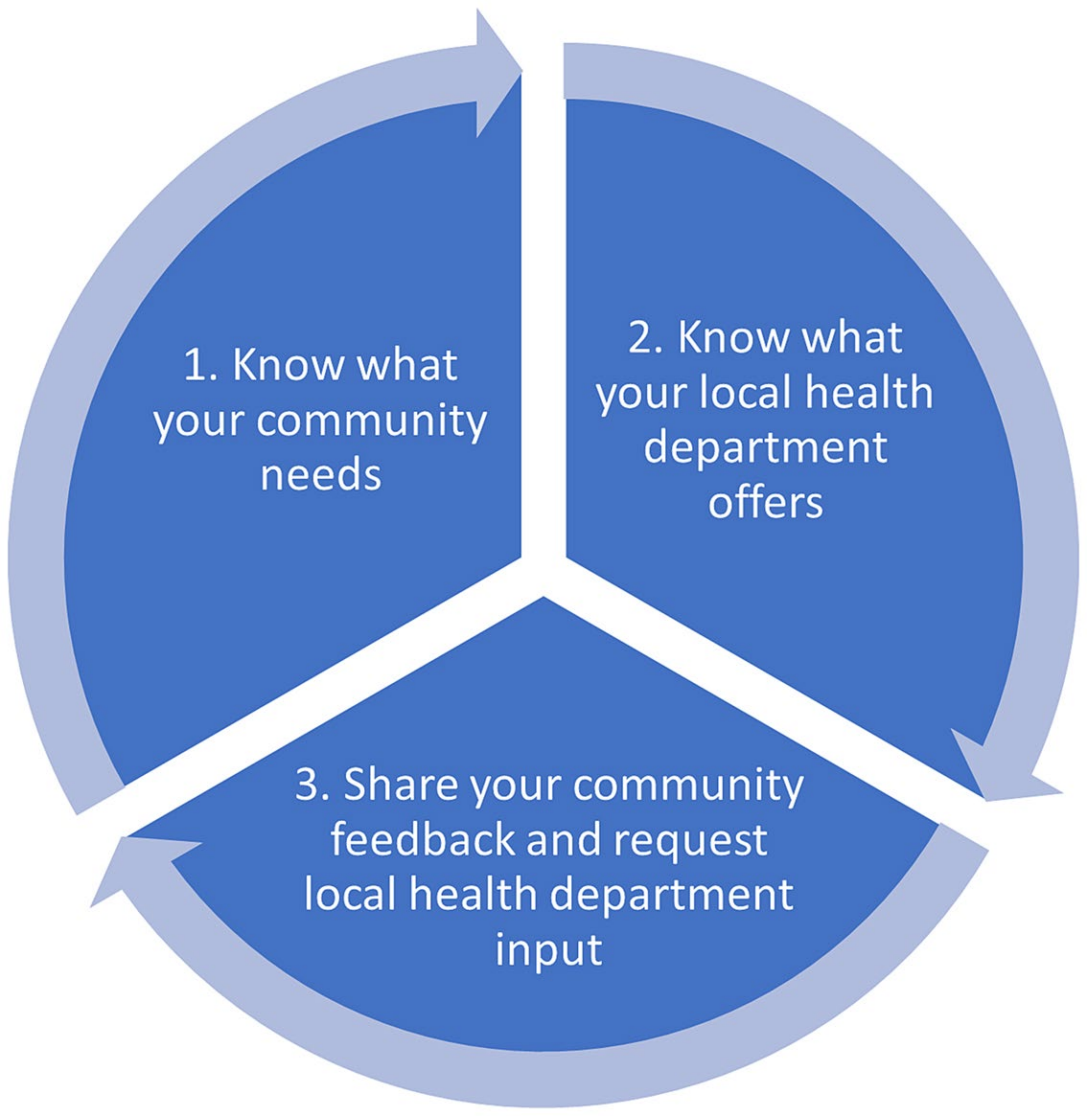
Three-fold approach to COVID-19 advocacy efforts.

This case study focuses on RICE’s advocacy efforts and relationship building with the ACPHD. Figure 1 illustrates three main advocacy-related activities that occurred simultaneously.

### 3.1. Activity 1: community needs assessment survey: know what your community needs

Since there was limited COVID-19 data available that included preferred language, RICE conducted a community needs assessment survey to learn directly from LEP individuals’ lived experiences. In September 2021, KCCEB reviewed existing surveys and selected questions from Universiti Malaysia Sarawak’s COVID-19 vaccine survey (26). These questions were adapted to add contextually-relevant vaccine hesitancy factors for immigrants and refugees in the United States, yielding 16 open-ended and closed-ended questions. Open-ended questions asked respondents to elaborate on the most difficult part of the pandemic and their sense of safety or perceived risk amidst rising hate incidents. Closed-ended questions had multiple-choice options and covered COVID-19 testing, trusted sources of COVID-19 information, and COVID-19 vaccination. Bilingual, bicultural community health workers from each RICE organization, who come from the communities they serve, translated the English survey into one or more of the other 15 languages. These health workers also validated the content to ensure that the survey’s messaging was accurate and relevant for their specific communities (9, 21, 27). The survey was offered in both paper and online forms.

Survey respondents were selected through convenience sampling, using RICE’s client lists and community events for recruitment. Individuals aged 18 or older who participated in RICE community events, COVID-19 outreach, and vaccination events were eligible for the survey. From October 2021 to February 2022, RICE administered the survey in three cities: Oakland, San Leandro, and Alameda. In administering the survey, the bilingual and bicultural community health workers provided support in respondents’ preferred languages and offered incentives, such as safety care kits with masks, test kits, sanitizer, safety whistles and alarms, and opportunities to win prizes like a gift card for a local Asian supermarket. There were 559 respondents across 12 languages: Amharic, Arabic, Burmese, Chinese, English, Farsi, Khmer, Korean, Mam, Nepali, Tigrinya, and Vietnamese.

After data collection, KCCEB used descriptive statistics to calculate frequencies and percentages for survey items. KCCEB compiled and shared survey findings with RICE members for feedback before a final analysis was completed in April 2022. The survey findings were then used to adapt RICE’s COVID-19 education and vaccine outreach and inform advocacy efforts with the ACPHD.

### 3.2. Activity 2: health department landscape assessment: know what your local health department offers

Between November 2021 and October 2022, KCCEB conducted an ongoing “landscape assessment” of the ACPHD’s materials to better understand how the ACPHD’s COVID-19 response plan accounted for LEP communities’ needs over time. This type of assessment resembles the landscape analysis method in García et al. (28) but reverses the roles of studier and studied by having a community organization assess the local health department’s impact. In this case, KCCEB reviewed the ACPHD’s COVID-19 website, including webpages, printable resources, fact sheets, and vaccine appointment platforms, to assess for language accessibility. At the time of this review, the ACPHD’s COVID-19 website was available in multiple languages through the Google Translate function and offered downloadable resources in different languages. However, KCCEB identified issues including translation errors, inconsistent availability of languages across resources, and lack of timely resource dissemination. For instance, some vaccine appointment questionnaires contained translation errors and the number of translations available for printable resources was inconsistent across the website.

Throughout this period, KCCEB invited ACPHD officials to RICE’s monthly meetings to share bilateral feedback and to better understand the ACPHD’s structure and translation process. Through informal conversations, RICE shared findings from the ACPHD website review and community feedback on the language accessibility of COVID-19 services and resources, while gathering information about the ACPHD’s process. ACPHD officials engaged in constructive dialog with RICE about the ACPHD’s practices and shared the website review findings with their teams for correction. In addition, ACPHD officials provided county COVID-19 updates relevant for RICE’s outreach and vaccine work and invited RICE to advisory boards to provide community feedback on language access and other public health issues.

Prior to the landscape assessment, RICE was unfamiliar with the ACPHD’s language equity practices and did not yet have an advocacy plan. Through the iterative process of gathering data in meetings with ACPHD officials and by reviewing the ACPHD’s COVID-19 website, RICE: (1) created a list of questions that served as a starting point to understand the ACPHD’s language equity infrastructure (Appendix 1); (2) identified strengths and areas for improvement in the ACPHD’s COVID-19 response plan for LEP communities; (3) learned what considerations guide the ACPHD’s decision-making process; and (4) developed data-informed advocacy requests across three domains of visibility, oversight, and funding.

### 3.3. Activity 3: relationship building between community organizations and local health departments: share your community feedback and request local health department input

In the course of relationship building between RICE and the ACPHD, KCCEB utilized power mapping tools to identify champions within the ACPHD. KCCEB defines a *champion* as an individual who is committed to collaborating toward a shared mission, has access to decision makers, and is willing to leverage their influence to share information and connect community organizations with key players. By identifying and connecting with champions, KCCEB facilitated the relationship between RICE and the ACPHD throughout the project.

Before the grant, KCCEB established its relationship with the ACPHD by identifying and engaging with champions in the ACPHD’s Community Assessment, Planning, and Evaluation unit to understand COVID-19 hotspot neighborhoods across ethnolinguistic groups and receive a letter of support for the grant project from the ACPHD leadership. These efforts allowed RICE to: (1) focus on hotspots with the highest COVID-19 cases and lowest vaccination rates; and (2) establish direct relationships with individuals and departments who were committed to working with RICE.

KCCEB invited champions to RICE meetings to learn about the ACPHD’s COVID-19 language access efforts, obtain COVID-19 testing and vaccination data from the county, share concrete community feedback, and ask how community organizations could collaborate with the ACPHD. In return, KCCEB was invited to and consistently attended the ACPHD’s biweekly health equity meetings and co-founded a community organization-led working group with ACPHD officials. As a result, KCCEB and ACPHD officials streamlined their communication from formal email exchanges to phone calls and in some cases text messages. In addition, KCCEB and RICE organizations started attending the Greater Bay Area Collective Impact Table hosted by the California Department of Public Health as part of its *Vaccinate All 58* Campaign (California’s campaign to equitably vaccinate all 58 counties) (29). Through these meetings, KCCEB bridged region-specific COVID-19 information between LEP communities and the California Department of Public Health. By communicating regularly with the county and state, KCCEB and RICE shortened the feedback loop between health departments and LEP communities.

Since most RICE organizations are primarily direct service providers, KCCEB organized an advocacy training series to equip RICE with skills to advocate with ACPHD officials. In May–July 2022, KCCEB contracted Asian Pacific Environmental Network to conduct a three-part advocacy training for RICE to determine its long-term language access recommendations for the ACPHD. During these sessions, RICE learned about power mapping, power strategies, and campaign development. RICE integrated these tools with its community needs assessment survey and landscape assessment findings to further build its advocacy campaign regarding LEP visibility, oversight, and funding.

In early July 2022, KCCEB met with the ACPHD’s COVID-19 Division to formally introduce RICE, share the community needs assessment survey and landscape assessment findings, and provide language access recommendations. Later in the month, RICE was invited by the California Department of Public Health *Vaccinate All 58* staff to introduce its COVID-19 work, becoming the first group of community organizations to meet with the California Governor’s new Office of Community Partnerships and Strategic Communications. Through this process, KCCEB, RICE, the ACPHD, and the California Department of Public Health moved toward engaging in mutually beneficial and reciprocal relationships with opportunities to hold each other accountable for public health language equity work.

## 4. Evaluation

KCCEB conducted the final evaluation of RICE’s COVID-19 interventions in November 2022. This section describes the results of the three activities, individually and collectively.

### 4.1. Activity 1: community needs assessment survey

**Figure 2:**
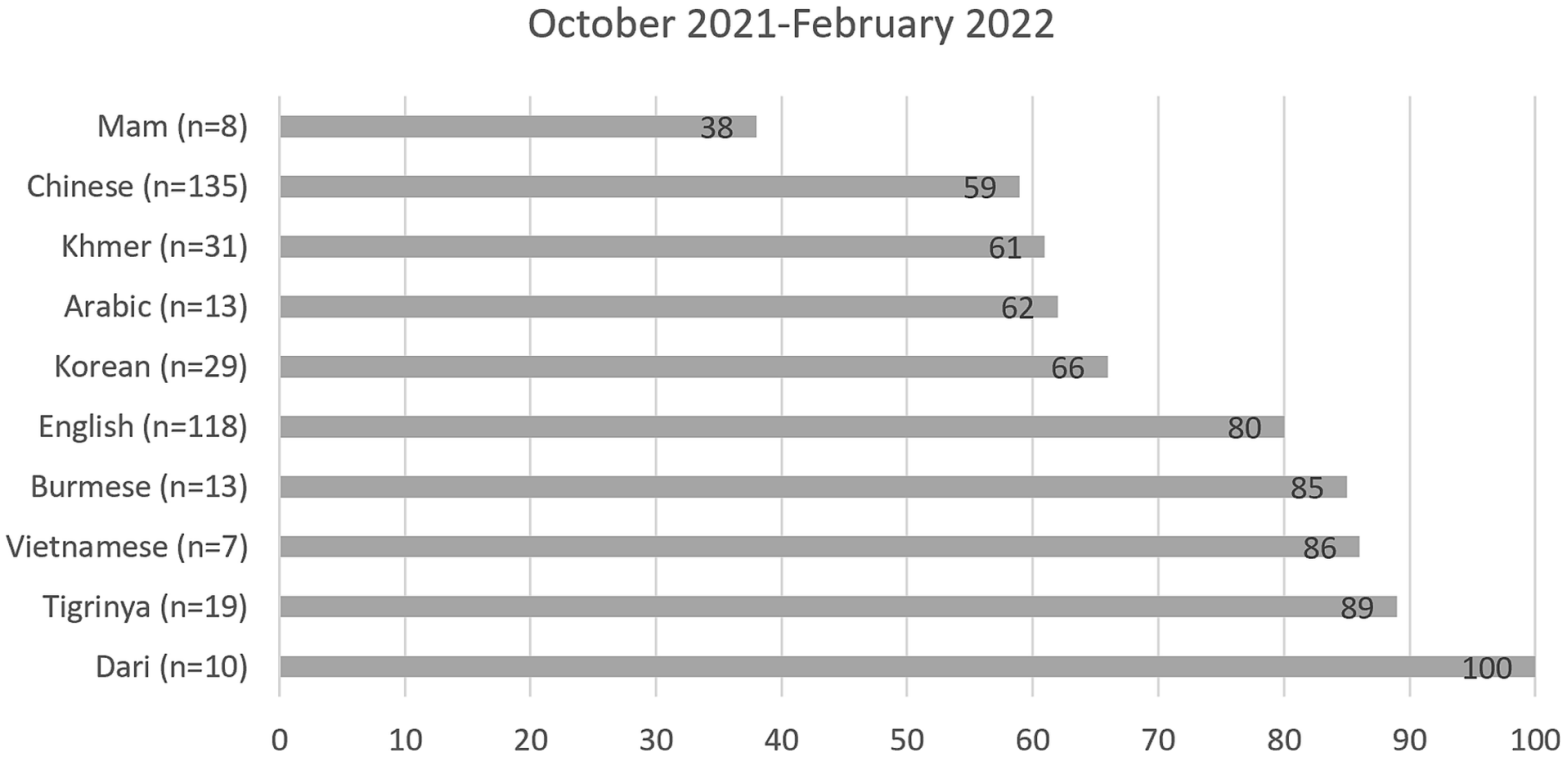
Percentage of respondents who reported ever being tested for COVID-19, by language group.

RICE’s survey showed important similarities and differences across LEP subgroups. Findings are shown in Figure 2, Tables 1, 2. In each table, the sample size (*n*) represents the number of respondents answering that question.

**Table 1:** Top two sources of COVID-19 information reported as most reliable, by language group.

	Friends, family, and neighbors	Social media	Mass media	Health care workers	Community and religious leaders
Arabic (*n* = 12)	2	1			
Burmese (*n* = 10)		1	2		
Chinese (*n* = 166)		2	1		
Dari (*n* = 10)	2	1			
English (*n* = 152)			2	1	
Khmer (*n* = 31)	1				2
Korean (*n* = 28)			1		2
Mam (*n* = 8)	2=	1		2=	
Tigrinya (*n* = 19)	2		1		
Vietnamese (*n* = 8)	2			1	

1: source rated as most reliable. 2: source rated as second most reliable. =: the same number of respondents picked these answers.

**Table 2:** Top two reported reasons for getting COVID-19 vaccine, by language group.

	To protect loved ones	To avoid getting COVID-19	To meet a requirement for work or school	To meet a requirement for travel	To resume normal life without masks	Because family or friends asked me to
Arabic (*n* = 13)		1	2			
Burmese (*n* = 10)	1	2				
Chinese (*n* = 160)	1				2	
Dari (*n* = 10)			2	1		
English (*n* = 159)	1	2				
Khmer (*n* = 29)	2	1				
Korean (*n* = 28)	2	1				
Mam (*n* = 9)	2=	1	2=			2=
Tigrinya (*n* = 19)	1					2
Vietnamese (*n* = 9)	1=				1=	

1: most important reported reason for getting vaccinated. 2: second most important reported reason for getting vaccinated. =: the same number of respondents picked these answers.

Figure 2 shows the percentage of respondents who reported having been tested for COVID-19. Table 1 presents respondents’ most trusted sources for COVID-19 information. Table 2 shows respondents’ reasons for getting a COVID-19 vaccine. RICE’s needs assessment survey also asked about the perceived reasons that friends and family may have opted not to get vaccinated. Across all 10 language groups, respondents most commonly chose “Vaccine has side effects/not safe” and “Not sure if vaccine is effective/not enough research.” Finally, the survey included a question about perceived safety or risk in the context of COVID-19 and rising hate incidents, and found differences across the language groups. In the four largest groups, respondents answering in Chinese (59%, *n* = 123) reported feeling unsafe during daily activities more than those answering in English (24%, *n* = 129), Korean (20%, *n* = 20), or Khmer (18%, *n* = 22).

By revealing differences across LEP subgroups, these survey findings demonstrate the importance of collecting and reporting data disaggregated by preferred language to more accurately capture LEP communities’ experiences. RICE utilized these findings to identify LEP communities needing better support and to adapt testing, education, and outreach strategies to serve these communities more effectively. For example, the survey findings indicated that specific respondent groups (Chinese and Khmer) had significantly lower COVID-19 testing rates than respondents answering in English. This level of detail would have been obscured had the survey utilized the monolith “Asian” category. In addition, these disaggregated findings strengthened RICE’s data-driven advocacy requests to the ACPHD.

### 4.2. Activity 2: health department landscape assessment

**Figure 3:**
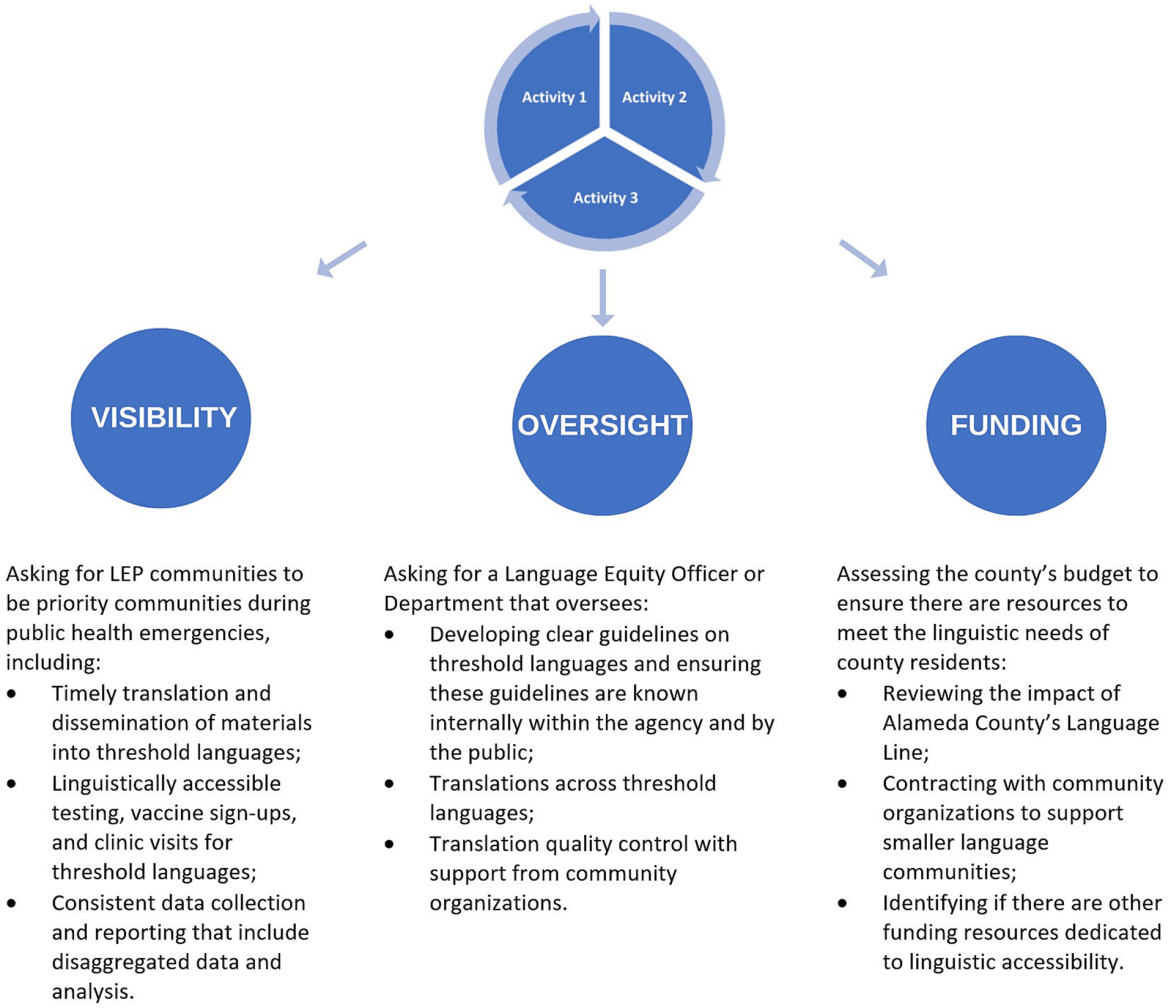
RICE’s development of advocacy requests.

Figure 3 illustrates the interactive process for transforming the results of the three activities into visibility, oversight, and funding advocacy requests to the ACPHD. This process was led by RICE and realized as recommendations from RICE to the ACPHD, rather than as a model shared between RICE and the ACPHD. RICE’s oversight questions revealed that the ACPHD had no language equity point-person or department responsible for overseeing communications and translations. RICE’s campaign therefore prioritized recommending that the ACPHD adopt an officer or department responsible for ensuring that public health services and communications be accessible, timely, high quality, and culturally and linguistically appropriate. Ultimately, RICE’s data-informed requests were critical in guiding conversations and creating a partnership with ACPHD officials to co-create solutions.

### 4.3. Activity 3: relationship building between community organizations and local health departments

RICE built relationships with ACPHD officials to shorten the feedback loop between local health departments and LEP residents and to include LEP community voices in decision-making. RICE’s work has directly impacted ACPHD guidelines and the California Department of Public Health’s language equity funding. After KCCEB’s presentation with the ACPHD’s COVID-19 Division, RICE’s language equity recommendations were incorporated into the ACPHD’s 2022–2024 COVID-19 Strategic Plan that guides future pandemic response planning. Additionally, the ACPHD is including these recommendations in its internal planning document focused on engaging Alameda County Health Care Service Agency leadership and community partners around language access, which impacts several important county departments.

Furthermore, the California Department of Public Health *Vaccinate All 58* Campaign’s Neighborhood Partnership Program, a statewide COVID-19 prevention mini grant, was expanded to include funding for language translation and interpretation as a direct result of RICE’s meeting with state officials. This increased access to COVID-19 outreach and vaccine events and resources for both LEP communities and community organizations across California. In this way, RICE facilitated grassroots community organizations’ involvement in future public health planning. KCCEB, RICE, the ACPHD, and the California Department of Public Health are deepening into reciprocal relationships, in which the flow of exchange is multidirectional from community members (upward), community organizations (horizontal), and the local and state health departments (downward).

### 4.4. Importance of engaging in 3 activities together

While each activity yielded significant results, the three together were complementary and mutually reinforcing, multiplying the impact of KCCEB and RICE’s work. Through the community needs assessment survey and the landscape assessment, RICE positioned itself as a key player who had data highlighting gaps between LEP community needs and the ACPHD’s COVID-19 response plan. Only community organizations known and trusted by LEP communities could have gathered this data. Concurrently, RICE opened information-sharing pathways by prioritizing consistent and mutually beneficial relationship-building with ACPHD champions. By gathering comprehensive data and acting as a trusted bridge between LEP residents and the ACPHD, RICE engaged in thought partnership with ACPHD officials. Together, RICE and the ACPHD co-created organizational policy and guideline solutions, thereby meeting community needs within the ACPHD’s capacity.

## 5. Discussion

This section discusses the significance of RICE’s work vis-à-vis data disaggregation, language equity advocacy, and cross-sector collaboration.

To understand the impact of public health emergencies on LEP communities, public health agencies need to collect and report data disaggregated by variables such as language, race/ethnicity, and country of birth. Disaggregated data enables providers and researchers to more accurately identify health disparities, plan specific interventions for disproportionately affected subgroups of the population, and review subgroup-specific health outcomes (30, 31).

In contrast, aggregate data obscures trends in health behaviors and outcomes. For example, a 2020 Kaiser Family Foundation study of about 50 million patients found that Asian-American, Black, and Hispanic patients had higher rates of transmission, hospitalization, and death due to COVID-19 compared to White patients. Indeed, Asian Americans had the highest risk of hospitalization and death after testing positive for COVID-19 (32). Meanwhile, a 2020 scoping review by the National Academies of Sciences, Engineering, and Medicine did not identify Asian Americans or any Asian-American subgroup as one of the populations “with higher rates of severe morbidity, mortality, and transmission” due to COVID-19 (33). In the San Francisco Bay Area, another study of individuals tested for COVID-19 in 2020 found that Asian Americans had the highest hospitalization and mortality rates among the four largest racial/ethnic groups (34). These seemingly contradictory findings make it challenging to understand the effects of COVID-19 on Asian American subgroups in 2020, as they estimate cumulative or “average” effects over heterogeneous demographic categories.

The way we analyze population health data affects how we understand outcomes and allocate resources to reach underserved populations (33). RICE’s survey did not gather clinical data, but by collecting disaggregated data about behaviors and attitudes RICE learned that LEP subgroups in Alameda County had different rates of COVID-19 testing, trusted sources of information, and reasons for getting vaccinated. These findings informed RICE’s direct services and advocacy, leading to more equitable, linguistically and culturally responsive interventions.

For example, RICE’s COVID-19 needs assessment revealed that LEP communities receive public health information differently depending on the language, format, source, and medium through which the message is delivered. This finding confirmed what the RICE organizations knew from years of direct service: one-size-fits-all approaches to communication (e.g., posting information on a website; sending mass mailings, emails, or texts; using only one communication platform or channel) are far less effective than diversified approaches (e.g., translating and tailoring messages for specific audiences; engaging directly with community members in person or on preferred social media/messaging apps; enlisting community leaders to deliver messages) (35). Gathering more disaggregated behavioral, attitudinal, and clinical data in the future would presumably illuminate further differences across LEP subgroups and allow community organizations, public health agencies, and health systems to better develop tailored interventions.

Previous studies argue that although there is little available data about LEP groups’ communication experiences during the COVID-19 pandemic, LEP groups for whom such data exists were adversely affected by the limited availability of timely, accurate information from trusted sources in languages other than English (36, 37). RICE’s way of engaging with communities and the ACPHD may provide a useful model of how collecting and reporting disaggregated community data can play a part in raising community voices, involving community members in decision-making, and co-developing interventions that recognize communities’ needs and assets. RICE’s strengths-based model of advocacy and partnership is consistent with Chipman et al.’s public health ethics framework for LEP populations (38) and ultimately highlights the need for public health departments to collect similarly detailed data.

From its formation, RICE prioritized advocating for language equity. The partners found framing issues around language more effective for LEP communities than focusing solely on racial/ethnic disparities in each community. By tackling language inequity, RICE addressed language itself as a social determinant of health (38–40) and built bridges across diverse communities. Working as a multiracial, multilingual collaborative also created strength in numbers and proved especially beneficial for RICE’s smallest LEP communities, who often had the least access to public health information in their preferred languages. A key takeaway from RICE’s COVID-19 pandemic response work is that partnerships between community organizations and public health departments are critical for (1) facilitating LEP communities’ access to services and (2) encouraging systemic changes in the public health emergency framework to ensure communities’ needs are visible and well-resourced. Throughout the pandemic, the RICE organizations provided their own community-specific services and advocated for better language access.

The Public Health 3.0 Framework calls for cross-sector collaboration–between public health authorities, working as Chief Health Strategists, and a range of community partners, who need to be appropriately funded and involved in shared decision-making (13). While there is a growing body of research on such collaborations, there is still no consensus on how to include community voices in these projects (41, 42). A review by Petiwala et al. suggests that “passive” strategies like gathering community data are necessary but less empowering for community members than “active” strategies that entail relationship-building, collaboration, and ultimately shared decision-making with communities (42).

The RICE organizations, composed of individuals from the communities they serve, pursued an approach to community assessment, advocacy, and services that facilitated meaningful collaboration between communities and public health. We argue that RICE’s approach of gathering data disaggregated by preferred language, and then strategically using this data to advocate for LEP communities and inform public health decisions, made gathering data an “active” step in a larger strategy.

Public health departments benefit from partners like RICE because these community organizations provide access to social networks, interpret complex community needs, and relay community priorities–all of which help public health departments improve health outcomes and health equity in the communities they serve. As Đoàn et al. (11) point out, community organizations, which often have a track record of working with marginalized communities and earning their trust, are particularly well positioned to support public health in crises like the COVID-19 pandemic (11).

## 6. Methodological constraints and strengths

This case study and the community needs assessment survey described within it have constraints and strengths worth noting. First, this case study examines in depth the community organization perspective of the partnership rather than the health department perspective, as it was co-written by KCCEB staff and the study design had no provision for formal interviews and surveys with health department officials. The purpose of the study was to present the work that community organizations do to gain and maintain community trust, advocate for marginalized, linguistically diverse communities, and devise constructive ways to partner with local health officials. Second, RICE recruited respondents for the community needs assessment survey through convenience sampling at community vaccine events, meaning respondents were already connected to community organizations and motivated to get the vaccine. Still, COVID-19 testing rates differed across LEP subgroups even in this sample, suggesting that testing rates would still be lower among harder-to-reach respondents. Third, RICE’s data collection involved multiple methods, as each organization collected data differently to fit its community. While it would have been methodologically streamlined to apply a single method, the community organizations recognized that a “one-size-fits-all” approach would limit accessibility. By asking respondents how they preferred to take the survey, the RICE organizations strove to make research a more accessible, positive experience for community members. Fourth, RICE collected only categorical and narrative data in the community needs assessment. No multivariate analyzes were conducted. Since descriptive data and studies are generally extremely limited for diverse language communities, RICE’s primary objective was to formulate–with the ACPHD–useful COVID-19 prevention and intervention guidance for each community. Lastly, the survey had differing sample sizes across language groups, with some sample sizes too small to generalize. Nevertheless, this case study confirms the importance of examining differences related to language and culture and shows how gathering disaggregated data might strengthen public health interventions and play a role in partnership-building.

## 7. Conclusion

We present this case study of KCCEB and RICE’s language equity work to invite public health departments and community organizations to consider key elements of effective advocacy, partnership, and mutual accountability. It is also important for potential partners considering similar cross-sector collaborations to recognize the vital role of flexible and sustainable funding (43, 44) as well as frequent communication and coordination between all stakeholders.

A NACCHO grant enabled KCCEB and RICE to strengthen existing relationships among seven community organizations, the ACPHD, the California Department of Public Health, and county residents. Although the RICE organizations had worked together before, this grant allowed the organizations to formalize a working relationship with the ACPHD and provided the RICE organizations with much-needed funding for all the additional COVID-related work they were doing. With county resources being limited at the beginning of the pandemic, organizations focusing on Asian languages or smaller linguistic communities received modest funding directly from the local public health department. Some organizations, like the ones in RICE, pursued state- and federal-level funding sources to meet community needs.

Building and maintaining a multilateral, cross-sector partnership required effective communication and coordination. The RICE organizations and the ACPHD, each interacting with their own clients and stakeholders under different organizational constraints, needed to work to stay in step with each other. The community organizations, being smaller, more structurally flexible, and more regularly in contact with clients, could quickly pivot when they learned of new needs. A recurring challenge was that the ACPHD, responsible for overseeing the whole county’s public health, could not always respond as quickly. The RICE organizations learned to pace their communications with the ACPHD accordingly, while maintaining consistent contact.

RICE worked sustainably by leveraging each partner’s strengths and capacity. The seven community organizations communicated regularly via texts, phone calls, and emails, ensuring that each partner’s circumstances and perspectives were understood. In addition, RICE intentionally pooled resources like personal protective equipment and safety kits to help the organizations fill shortages they occasionally faced. Sharing supplies not only provided better services to communities, but also strengthened trust and goodwill between the organizations, facilitating further collaboration.

KCCEB and RICE are continuing their language equity advocacy work with the ACPHD. During 2023–2024, RICE is conducting a systems-level needs assessment with the ACPHD (based on the questions in Appendix 1) to identify and address root causes of the public health department’s language access gaps. With these findings, RICE plans to make language equity recommendations to the ACPHD, advocating broadly on behalf of local LEP communities.

## Data availability statement

The raw data supporting the conclusions of this article will be made available by the authors, without undue reservation.

## Author contributions

DK led the writing and analyses. AL coordinated the interventions and assisted with the writing. ED-H assisted with the writing and conceptualization of the study. DA was team lead and assisted with the writing. All authors contributed to the article and approved the submitted version.

## Appendix

**Table:**

RICE’s Language Equity Questions for the ACPHD
VISIBILITY What is the county’s plan for collecting and reporting disaggregated population data and/or linguistic data for public health emergencies (i.e., COVID-19 testing, vaccination, etc.)? What are the threshold languages in the county? Are these threshold languages standardized throughout the county’s system or is it different depending on the department (i.e., Local Health Department, Behavioral Health, Environmental Health, etc.)? Where can we locate the list of official threshold languages? What is the percentage used to determine “threshold languages”? OVERSIGHT What are the current county guidelines and policies around language equity? How does the county assess its own compliance and adherence to these policies and guidelines? Within the ACPHD, who and/or which department is responsible for managing language translations? How does the ACPHD and/or each department determine which materials will be translated? What is the material translation process for the ACPHD? Will the translated material go through a review process to ensure accuracy? Who makes decisions on how health information is advertised, promoted, and disseminated? FUNDING Where is the county currently investing its language equity resources? Has the county already assessed the impact and effectiveness of its language equity resources and if not, how can the county take steps to make this assessment? How can community organizations provide concrete suggestions on how the county can improve where it invests its dollars for language equity resources? How is the target community’s expertise used and compensated for planning material translation? What are ways that the county can fund organizations who are already doing interpretation, linguistic and cultural translation work?
